# The Role of Regional Therapies for in-Transit Melanoma in the Era of Improved Systemic Options

**DOI:** 10.3390/cancers7030830

**Published:** 2015-07-01

**Authors:** Emmanuel Gabriel, Joseph Skitzki

**Affiliations:** 1 Department of Surgical Oncology, Roswell Park Cancer Institute, Buffalo, NY 14263, USA; E-Mail: emmanuel.gabriel@roswellpark.org; 2 Department of Immunology, Roswell Park Cancer Institute, Buffalo, NY 14263, USA

**Keywords:** in-transit melanoma, regional cancer therapy, immunotherapy

## Abstract

The incidence of melanoma has been increasing at a rapid rate, with 4%–11% of all melanoma recurrences presenting as in-transit disease. Treatments for in-transit melanoma of the extremity are varied and include surgical excision, lesional injection, regional techniques and systemic therapies. Excision to clear margins is preferred; however, in cases of widespread disease, this may not be practical. Historically, intralesional therapies were generally not curative and were often used for palliation or as adjuncts to other therapies, but recent advances in oncolytic viruses may change this paradigm. Radiation as a regional therapy can be quite locally toxic and is typically relegated to disease control and symptom relief in patients with limited treatment options. Regional therapies such as isolated limb perfusion and isolated limb infusion are older therapies, but offer the ability to treat bulky disease for curative intent with a high response rate. These techniques have their associated toxicities and can be technically challenging. Historically, systemic therapy with chemotherapies and biochemotherapies were relatively ineffective and highly toxic. With the advent of novel immunotherapeutic and targeted small molecule agents for the treatment of metastatic melanoma, the armamentarium against in-transit disease has expanded. Given the multitude of options, many different combinations and sequences of therapies can be offered to patients with in-transit extremity melanoma in the contemporary era. Reported response and survival rates of the varied treatments may offer valuable information regarding treatment decisions for patients with in-transit melanoma and provide rationale for these decisions.

## 1. Introduction

The incidence of melanoma has steadily risen in the United States since the 1970s, with an estimated 76,000 new cases in 2014 [1]. Following surgical excision of the primary, recurrent disease can occur locally, regionally, or distantly. In-transit (IT) disease refers to metastases within the regional dermal and subdermal lymphatics near the primary melanoma but distal to the draining regional lymph node basin [2]. Overall, approximately 4%–11% of melanoma recurrences present as IT metastases [3,4,5,6,7]. Several factors are associated with increased risk for IT metastases. These include patients with thicker primary melanomas (>1 mm), tumor ulceration, lymphovascular invasion (LVI), regional nodal involvement and location of the primary on a lower extremity [3,7,8,9]. The rates of IT disease vary substantially in the presence of these other factors, with higher incidences associated with more aggressive features.

Based on the 2010 7th edition of the American Joint Committee on Cancer (AJCC) Cancer Staging Manual, IT disease is categorized within the nodal staging of the TNM staging system [10]. The presence of IT metastases correlates with clinical stage III melanoma, which is further divided within pathological staging into IIIB and IIIC, depending on primary tumor ulceration and whether lymph node metastases are concurrently involved (IIIB does not have lymph node metastases, whereas IIIC does). The prognosis of these stages varies but overall is quite poor, with reported five-year overall survival rates ranging from 20% to 60% [3,10,11,12,13]. IT disease itself is a risk factor for subsequent regional lymph node or metastatic disease, with up to 75% of patients with IT disease developing nodal or distant metastases [3,6]. Consequently, patients with IT disease should be carefully evaluated for additional sites of disease and undergo a detailed history and physical examination with scrutiny to the lymph node basins as well as staging imaging studies. The unfavorable outcomes and risk of advanced melanoma associated with IT disease reflect the relevance and need for effective therapies for these patients. In this review, the available treatments for IT melanoma of the extremity and their relative efficacy are discussed. With the emergence of improved systemic therapies, the role of regional therapies in comparison with newer, immunotherapeutic and targeted molecular agents are examined. Based upon the published data, treatment algorithms and justification for these treatment approaches are detailed.

## 2. Treatments for IT Melanoma

A wide range of options is available for IT melanoma. The most recent guidelines from the National Comprehensive Cancer Network (NCCN) outline these options (Table 1), which are similar for both IT disease that is synchronous with the primary tumor and recurrent IT disease [14]. Options include surgical resection, intralesional and topical treatments, local radiation, regional therapies (comprised of isolated limb infusion (ILI) and isolated limb perfusion (ILP)), and systemic agents (including chemotherapies, interferon (IFN) and newer immunologic drugs, such as anti-CTLA4 and anti-PD-1 antibodies) [15,16,17]. Current clinical trials are also investigating multiple modality approaches, for example combining ILI with adjuvant ipilimumab (NCT01323517) or melanoma vaccines with ipilimumab (NCT00094653) [18,19]. As reflected by the NCCN guidelines, there is no consensus on the optimal approach [14]. Treatment for a specific patient with IT melanoma may benefit from a multidisciplinary discussion to develop an individualized plan [20]. When evaluating the options, a global understanding of the benefits and shortcomings of each approach should be considered (Table 2).

**Table 1:** Treatment Options for In-transit Melanoma.

**Local therapies**	Surgical excision to clear margins
	Intralesional injection (BCG, IFN, IL-2, oncolytic viruses)
	Local ablation therapy
	Topical imiquimod
	Radiation therapy
**Regional therapies**	Isolated limb perfusion with melphalan
	Isolated limb infusion with melphalan
**Systemic therapies**	Immunotherapies
	Small molecule inhibitors
	Chemotherapies

**Table 2:** In-transit Melanoma Treatment Variables.

	Local Therapies	Regional Therapies	Systemic Therapies
**Systemic Toxicity**	↓↓↓	↓↓	↑↑
**Local Toxicity**	↑	↑↑	↓↓
**Technical Feasibility**	↑↑↑	↓↓	↑↑
**Repeat Potential**	↑↑↑	↑	↑

Number of arrows express relative degree: ↑ = yes; ↓ = no.

## 3. Surgery

Surgical resection to negative margins is considered the gold standard for IT melanoma. Unlike wide local excision (WLE) of the primary melanoma, 1–2 cm margins are not indicated as long as negative margins are obtained because IT disease already represents invasion into the subdermal lymphatics. This is a technically feasible option when the IT disease is limited to a single or few lesions, allowing for resection and subsequent closure of the wounds. However, in cases where there are multiple, widespread IT lesions along an extremity, surgical resection may not be appropriate as it may result in serious morbidity or significant deformity.

As noted earlier, IT disease is associated with an increased risk of nodal metastases. Some retrospective studies have reported an increased risk of IT disease following sentinel lymph node biopsy (SLNB) [21,22]. However, results from the prospective Multicenter Selective Lymphadenectomy Trial (MSLT-I) did not show a difference in the rate of either local recurrence or IT disease in patients with intermediate thickness melanoma who were randomly assigned to either observation or SLNB followed by completion lymphadenectomy in cases of a positive SLNB [23]. Other studies support the findings of MSLT-I in that SLNB itself does not increase the risk of IT metastases [24,25]. When IT disease presents as a recurrence, SLNB may be considered. Repeat SLNB is technically feasible. Studies have shown that about one-third of patients with IT disease and no clinical nodal disease (or macrometastases) were found to have positive SLNB, even though the SLNB was negative when initially biopsied during excision of the primary lesion [26,27]. While SLNB in this setting has no definitive survival benefit, it does offer the potential for more complete staging and further treatment with completion lymph node dissection (CLND) of the draining nodal basin for local control. However, the benefits of SLNB and CLND in patients with IT disease may not be outweighed by the risks of surgery, namely post-operative lymphedema, particularly in the setting of nodal micrometastases. As an alternative to immediate CLND in the setting of known positive SLNB, the eagerly anticipated results of the Multicenter Selective Lymphadenectomy Trial II (MSLT-II) may support the close surveillance of the nodal basin with routine ultrasound (US) followed by delayed CLND if a recurrence is detected [28]. Although patients with IT disease were excluded from MSLT-II, results of the trial may perhaps be extrapolated to this patient population since these patients already have stage N2c disease without macrometastases. Therefore, even in the presence of micrometastatic disease discovered from a SLNB, observation with close US may be a viable alternative to immediate CLND.

## 4. Lesional Therapies

Local lesional therapies include topical applications and intralesional injections [29,30]. Imiquimod is a Toll-like receptor agonist which generates antitumor activity through the induction of pro-inflammatory mediators [31]. It is the typical topical drug used for melanoma, and it most often used for the lentigo maligna subtype [32,33]. In the setting of IT disease, imiquimod has shown beneficial effects alone and in combination with other intralesional therapies, although these studies are quite small [34,35]. In a small series of three patients, each with greater than 15 IT metastases, imiquimod resulted in >90% regression of IT lesions in two of the patients [34]. Supplementation with intralesional interleukin-2 (IL-2) generated responses in the third patient. Another small series of nine patients with IT disease combined imiquimod with intralesional Bacille Calmette-Guerin (BCG) [35]. Five patients had complete regression and one had a partial response. Patients were followed for median period of 35 months, at which point there were no recurrences. However, long-term data past years is lacking.

Intralesional injections are mainly comprised of BCG, interferon-alpha (IFN-α) and IL-2. BCG has been tested for recurrent disease. A meta-analysis of 15 studies with intralesional BCG in the adjuvant setting for patients with stage III melanoma showed complete response in 19%, partial responses in 26% and improved survival in 13% [36]. Intralesional IFN-α has been tested in patients with advanced melanoma. While there are reported benefits in local responses, use of IFN-α has not been shown to be curative. In one of the largest studies of intralesional IFN-α, among 51 patients with metastatic melanoma who had a least one cutaneous metastasis, there were 24 (47.1%) complete or partial local responses [37]. There are several studies that have investigated intralesional IL-2 for IT disease. In a retrospective analysis of 31 patients treated with IL-2, 10 patients (32.3%) developed a pathological complete response (pCR) and 17/31 (54.8%) had a partial response [38]. Systematic reviews combining other small studies such as this one have shown good local control of IT disease, with up to 50% of treated patients achieving complete response [39,40].

A more contemporary, novel intralesional therapy is the small molecule PV-10, a drug containing 10% of the chemical rose bengal which is derived from a synthetic iodine dye [41]. While it has a wide range of medical uses, this drug has been shown to have chemoablative properties in several cancer types [42,43]. Cell lysis in turn propagates exposure of tumor-associated antigen and leads to tumor-specific T cell-mediated responses, which is thought to mediate regression in uninjected cutaneous lesions through a bystander effect [42]. Very small series have reported promising objective response rates in patients with metastatic melanoma [44,45]. A recent phase 2, multicenter, single arm trial of rose bengal in 80 patients with refractory stage III/IV melanoma showed an overall response rate of 51% and complete response rate of 26% [46]. The durability of these responses was reported as a median of 4.0 months, with 8% of patients disease-free after 52 weeks.

In general, side effects of the different intralesional therapies are well tolerated [39]. While this data appears impressive regarding the treated lesions, the ability of intralesional therapy to target microscopic IT lesions or deep deposits is limited. Therefore, the role of intralesional therapies often serves as an adjunct to more definitive treatments, and may function to palliate symptoms in patients who are too high risk to undergo more aggressive treatments.

## 5. Oncolytic Virus-Based Vaccines

Melanoma vaccines have long been sought as potential treatment options for systemic disease, although their use is currently limited to clinical trials and has not reached mainstream recommendations. Oncolytic viruses (OVs) have been developed to act through tumor tropic mechanisms resulting in direct oncolysis of tumor cells and subsequent antitumor events within the tumor microenvironment, potentiating systemic responses toward antitumor immunity [47]. Animal melanoma models investigating recombinant adenoviruses or vaccinia pox viruses have shown promising antitumor responses [48,49,50]. Translation of these virus-based strategies to human trials has shown up-regulation of anti-tumor immunity in the setting of good patient tolerability with minimal side-effects, although these trials have been very small phase I/II studies [51,52,53]. Clinical trials are on-going to characterize and develop more fully the application of melanoma vaccines into standard therapy as single agents or in combination with existing treatments, which potentially may prove effective for patients with IT disease [19,54,55].

One new and very promising oncolytic viral construct is the herpes simplex virus type-1 based immunotherapy designated Talimogene laherparepvec (T-VEC). This virus selectively targets tumor cells and results not only in lytic destruction but also overcoming systemic anergy to tumor associated antigen through exposure of these antigens and production of GM-CSF to recruit macrophages to the tumor microenvironment [56]. In the phase III OPTiM trial, T-VEC was compared to subcutaneous GM-CSF in patients with advanced melanoma where the primary endpoint was durable response rate (DRR) lasting at least six months. In the cohort, 30% had stage IIIB/C disease with the remainder having stage IV disease. DRR was reported in 16% of patients who received T-VEC compared to 2% of patients who received GM-CSF alone [57]. The objective response rate (ORR) with T-VEC was 26% with and 11% CR *versus* a 6% ORR and 1% CR with GM-CSF alone [57]. The therapy was well tolerated with the most common adverse events (AEs) being fatigue, chills and pyrexia occurring in 26% of the T-VEC group *versus* 13% in the GM-CSF alone group. Recent updates of this trial shows that OS has approached statistical significance with a median OS of 23.3 months with T-VEC *versus* 18.9 months with GM-CSF (HR = 0.787, 95% CI 0.62–1.00) [56]. In addition, T-VEC has been shown to decrease the size of primary tumors facilitating resectability. A subset analysis of 37 patients treated with T-VEC at the primary who then underwent surgery showed that six patients converted from inoperable to operable, and 15/37 showed no evidence of disease [58]. Further multimodal investigations with T-VEC are ongoing, including a phase Ib/II trial combining T-VEC with ipilimumab [59].

## 6. Radiation

Radiation therapy (RT) is not typically indicated for IT disease. Conversely, it is more often utilized in patients with high-risk local-regional recurrence, including patients with desmoplastic melanoma or high nodal disease burden [60,61,62,63]. However, RT has a beneficial role in the palliation of local symptoms, such as bleeding, pain and fungating infections, which may result from extensive IT disease [64,65,66]. While not curative, high RT doses (>30 Gy) have been shown to obtain longer disease-free survival rates and overall survival compared to patients treated with lower RT doses (≤30 Gy) [67]. RT is not without side-effects, which include short-term changes such as local edema, erythema, and desquamation as well as long-term risks such as fibrosis and later development of sarcoma [61,68]. Similar to lesional therapies, RT can be administered in an adjunctive or palliative role for patients with IT melanoma, although its use as a single modality treatment is minimally effective with the potential for significant toxicity.

## 7. Regional Therapy

Regional therapies for IT melanoma of the extremity include isolated limb perfusion (ILP) and isolated limb infusion (ILI). ILP has been used for extremity melanoma for over 50 years [69]. It is typically performed in the operating room under general anesthesia. The technique includes vascular dissection of the inflow and outflow vessels to the targeted extremity (such as the iliac or femoral vessels in the lower limb or the axillary vessels in the upper limb), large bore cannulation of the vessels under direct vision, application of a proximal pneumatic tourniquet, external warming of the extremity using heating blankets and circulation of warmed chemotherapy. Temperatures during ILP typically reach 42 °C [70,71]. However, ILI typically cannot reach this degree of hyperthermia with temperatures closer to 40 °C [72,73]. To ensure proper delivery and minimize systemic exposure to the circulating chemotherapy, the circuit is monitored for appropriate flow rates and absence of leakage [74,75]. A pump oxygenator can be used to maintain normal oxygenation and pH of the circulating perfusate. Because of the surgical dissection involved, this technique allows for lymph node removal as well. Once the perfusate is circulated through the affected extremity for 60 min, it is evacuated and discarded as the limb is further washed out with crystalloid solution. When the tourniquet is removed, all chemotherapy has been removed, so systemic toxicity should be negligible.

ILI is similar to ILP, but takes advantage of advancements in interventional radiology and percutaneous catheter placement. ILI is a minimally invasive approach which percutaneously accesses the diseased extremity inflow and outflow vessels [76]. The catheters typically have smaller calibers than those used in ILP, which results in slower infusion flow rates. Access is obtained through the contralateral groin in the case of lower extremity IT disease, whereby the catheters are directed up and over the aortic bifurcation. For upper extremity IT disease, a groin approach can also be utilized. Similar to ILP, a pneumatic tourniquet is applied, and the circuit is connected to a heating device. However, an oxygenator is not typically incorporated, which leads to higher levels of hypoxia and acidosis as compared to ILP [77]. As the circuit is hypoxic and acidotic, treatment duration is typically shorter for 30 min total. The hypoxic and acidotic aspects of ILI are thought to contribute to the antitumor effects of this treatment. Similar to ILP, ILI infusate is discarded and the limb washed out prior to removal of the tourniquet, which limits systemic toxicity. Since its development in the 1990s, ILI continues to evolve and offers a less invasive treatment model for testing new therapies [78].

An important advantage of ILP/ILI is that they allow for high doses of chemotherapy to be delivered and isolated to the involved extremity. This is particularly relevant in instances of diffuse IT disease and microscopic IT disease, which is technically not amenable to surgical resection. High dose, limb directed chemotherapy results in a greater treatment effect, while simultaneously minimizing systemic toxicity. The most commonly used chemotherapeutic agent in regional therapy is melphalan, a type of alkylating agent [79]. The dosage of melphalan for both ILP and ILI is volume based (7.5 mg/L lower extremity limb volume, 10 mg/L upper extremity limb volume) with ideal body weight correction for ILI. As previously stated, typical treatment durations are 60 min for ILP and 20–30 min for ILI [74,76]. Other agents have been investigated in conjunction with melphalan, including tumor necrosis factor-alpha (TNF-α), interferon-gamma (IFN-γ), actinomycin D, and ADH-1, which is a cyclic pentapeptide, that inhibits N cadherin expression important in melanoma cell adhesion and migration [80,81,82,83,84,85]. Additional agents, such as intra-arterial temozolomide which is an alkylating agent that is commonly used for systemic therapy of metastatic melanoma, has also been tested using an ILI model [86]. Results from this multicenter phase I study involving 28 patients showed a 10.5% complete response (CR) and 5.3% partial response (PR). An additional 3/19 (15.8%) patients had stable disease.

While there have not been prospective randomized controlled trials directly comparing ILP with ILI, several studies with long term follow-up have characterized the benefit of both of these regional therapies. Overall, ILP has been shown to have better local responses compared to ILI, albeit with increased risk of complications and toxicities [72]. A large U.S. retrospective review of 62 patients undergoing melphalan-based ILP and 126 patient undergoing ILI showed improved results for ILP [87]. The overall response rate (complete response [CR] + partial response [PR]) of ILP was 81% compared to 43% in the ILI group. ILP patients had a CR rate of 55% with a median duration of 32 months, whereas ILI had a CR rate of 30% with median duration of 24 months. A similar European study showed an overall response rate of 85% for ILP [88]. However, strong data is lacking regarding long-term benefit to survival outcomes. A Swedish study with 25-year follow-up compared ILP with WLE to WLE alone in a small number of patients (33 patients in the ILP + WLE group and 36 patients in the WLE alone group) [89]. Median melanoma-specific survival was 95 months for ILP + WLE group compared to 38 months for the WLE alone group, although this finding was not statistically significant (*p* = 0.24) as a result of the small sample size and low power of the study.

Further results of ILI have been reported from retrospective analyses. A large Australian study of 185 patients treated with ILI (using melphalan and actinomycin D) reported comparable local response rates to ILP, consisting of an overall response rate of 84%, with a median overall response duration of 13 months and 22 months for patients with CR [90]. The median overall survival was 38 months, but in patients with CR the median survival was 53 months (*p* = 0.005). It should be noted, however, that the median follow-up in this study was only 20 months. This group also performed a subset analysis based on age <75 or ≥75, with age-stratified CRs of 34% for age ≥75 years and 41% for age <75 (*p* = 0.28) [91]. The overall recurrence free interval after CR was 24 months and overall survival ranged from 36 to 39 months between the age groups, showing that more elderly patients derive similar benefit from ILI. A U.S. based study of 128 patients treated with melphalan and actinomycin D reported less beneficial outcomes compared to the Australian study, showing an overall response rate of 64%, CR rate of 31% and PR rate of 33% [92].

TNF-α has been studied in combination with melphalan in regional therapy. Several retrospective studies have reported 90% or better overall response rates [80,93,94]. However, in a direct comparison, combining TNF-α with melphalan has not been proven to have an additive response. The American College of Surgeons Oncology Group (ACOSOG) Trial Z0020 performed a prospective, multi-institutional trial of 133 randomized patients. Response rates were similar among the two treatment groups, with 64% in the melphalan-alone group and 69% of patients in the melphalan + TNF-α arm having a response to treatment at three months [95]. These results, however, remain controversial based on the types of assessments performed for tumor response. Addition of IFN-γ to melphalan and TNF-α has not been shown to have added benefit, either [82].

Multiple studies have characterized favorable patient-specific and tumor-related variables, which predict improved responses to regional therapy. The factors include female gender, low tumor burden, in-transit disease of the lower extremity, negative nodal disease, and complete response [96,97,98,99,100]. Unfortunately, even after obtaining CR, recurrences are common. One study following 36 patients with CR after ILP and 37 patients with CR after ILI reported a three-year recurrence rate of 65% and 85%, respectively [101]. Identification of patient-related or treatment-specific factors that can predict which patients will recur has proven difficult and continues to be the subject of ongoing interest [102]. For patients with in-field recurrence confined to the treated extremity, repeat ILP or ILI can be performed without significantly increased additional toxicity [103]. Surgical resection of residual disease following ILI can be performed, and offers some additive benefits. In a study of 176 patients with IT disease where 154 were treated with ILI alone and 22 patients with ILI and surgery, the former had a 68% PR, while the latter had a 34% CR and a 19% PR [104]. The ILI alone group had a median overall survival (OS) of 30.9 months, whereas the ILI + surgery group did not reach median OS, but this difference was not statistically significant (*p* = 0.304).

As discussed, there are several different options for regional therapy in IT melanoma, which offer the opportunity for local response and potentially cure. Both ILP and ILI, however, are associated with important, specific toxicities that must be taken into account before considering these treatments. A grading system developed by Wieberdink and colleagues characterized regional toxicities from no reaction (grade 1) to reactions that may necessitate amputation (grade 5) [105,106]. Because of the nature of these treatments, ischemia, muscle damage, compartment syndrome and subsequent renal insufficiency can result [92,107]. Long-term sequelae including lymphedema, paresthesias, pain and functional impairment may persist and negatively impact quality of life (QOL) [108]. Therefore, close post-operative monitoring is indicated following regional therapy. As a general reference for toxicity rates, the ACOSOG Z0020 trial showed grade 4 adverse events in 12% of 129 patients [95]. Retrospective studies have reported a range of grade 3 or higher toxicities from 18% to 36%, with ILI generally having less morbidity compared to ILP [72,92,109]. Amputation of the treated limb is a major complication of regional therapy, though fortunately most modern studies report that this occurs infrequently. For example, in the ACOSOG Z0020 trial, there were a total of three amputations among the 133 enrolled patients (2.3%) [95]. Retrospective studies have shown similarly low amputation rates [87,109]. It is important to note that amputation of the upper extremity may be more common than the lower extremity due to higher rates of regional therapy failure in upper extremity IT melanoma, as opposed to increased toxicity associated with the regional therapy [110].

## 8. Systemic Therapy

Historically, systemic chemotherapies and immunotherapies have been extensively studied and applied to metastatic disease. Chemotherapies include dacarbazine, temozolomide and others, while older immunotherapies include IFN and IL-2. Many of these agents have been investigated in both the adjuvant and neoadjuvant settings as a multimodality approach with metastasectomy [111,112,113,114]. However, these agents are associated with limited benefit for regional or distant disease in the context of significant toxicities.

Newer targeted agents have largely supplanted these therapies in the treatment of systemic disease as reflected in the most recent guidelines from the National Comprehensive Cancer Network (NCCN) [14]. These drugs are comprised of MAP kinase inhibitors, including vemurafenib, dabrafenib and trametinib; the anti-CTLA-4 inhibitor ipilimumab; and the anti-PD1 inhibitors lambrolizumab, pembrolizumab and nivolumab. Due to a more favorable side-effect profile compared to IL-2, these drugs have routinely become the first line treatments for distant metastatic disease, though their specific application to regional extremity IT melanoma is much less characterized and validated. Furthermore, these agents currently are not approved for use in the adjuvant setting following surgery, and so IFN-α remains the primary option for adjuvant therapy for involved regional lymph node disease. Therefore, it is challenging to directly compare the efficacy of the new immunotherapies to the more established treatments, such as ILP/ILI or lesional agents (Table 3). A detailed analysis of these agents for systemic disease is beyond the scope of this review. Thus in this section, we briefly discuss the benefits and side-effects of these recently approved agents.

There are two main targets in the MAP kinase pathway, which is involved in cell signaling and growth. Vemurafenib and dabrafenib inhibit the V600E mutant version of BRAF [115,116]. Approximately half of patients with melanoma express this gene mutation [117]. Vemurafenib has been shown to be superior to dacarbazine in a phase III randomized trial reporting an overall response rate of 48%, a median progression-free survival (PFS) of 5.3 *versus* 1.6 months and a one year OS rate of 55% *versus* 43% [118]. Dabrafenib is a more recently approved BRAF inhibitor with similar response rates. A phase III trial reported median PFS rates to be 5.1 months for dabrafenib compared to 2.7 months for dacarbazine [119]. Resistance to BRAF inhibitors is common, and disease progression often occurs after several months [120]. Trametinib is an inhibitor of MEK, which is a downstream signaling molecule in the MAP kinase cascade [121]. When used with dabrafenib, the combined BRAF and MEK inhibition has shown superior outcomes to vemurafenib alone. A phase III randomized trial showed that the one-year OS was 72% in the dabrafenib/trametinib group *versus* 65% in the vemurafenib group [122]. Additional data has shown that this combination has proven beneficial in patients who progress on first line BRAF inhibitors [123,124,125]. An update of one of these trials presented at the recent ASCO meeting reported improved one- and two-year OS in the combination group compared to dabrafenib alone [126]. The use of BRAF inhibitors as neoadjuvant therapy prior to surgical resection is also an area of interest, though there is limited data in this setting [127]. As a group, these agents are well tolerated with the main side effects including skin changes, nausea, fatigue, pyrexia and increased risk of squamous cell cancer [128,129,130].

**Table 3:** In-transit Melanoma Treatment Outcome Comparisons.

Treatment	OR	CR	PFS	OS
*Novel vaccines*				
T-VEC	26%	16%		Median 18.9 months
PV-10	51%	26%		
*Regional therapies*				
ILP	81%–90%	50%–82%	Median 6–26 months	Median 24–51 months
ILI	43%–84%	30%–44%		Median 31–53 months
*Systemic therapies*				
BRAF	48%–81%	1%–6%	Median 5.3–6.9 months	Median 13.6 months
				55% (1 year)
Ipilimumab	11%–29%	1.50%	Median 2.9 months	Median 10.1 months 47%–53% (1 year)
PD-1	26%–52%		Median 5.5 months 42.1% (1 year)	Median 8.2 months 69%–73% (1 year)

OR = Overall Response; CR = Complete Response; PFS = Progression-Free Survival; OS = Overall Survival.

As an immunotherapeutic agent, ipilimumab is a monoclonal antibody against CTLA-4, the expression of which is mechanistically associated with anergy to tumor-associated antigens [131]. In a phase III study, ipilimumab was compared to a peptide vaccine. Results showed superior OS when ipilimumab was used alone compared to the vaccine (median OS of 10.1 months *versus* 6.4 months), with no additive benefit of the vaccine (median OS of 10.0 months for ipilimumab + vaccine) [132]. Further studies have investigated ipilimumab in combination with a variety of agents, including chemotherapy and other melanoma vaccines [133,134,135]. A phase III study of ipilimumab plus dacarbazine showed superior five-year OS compared to dacarbazine alone (18.2% ipilimumab plus dacarbazine *versus* 8.8% dacarbazine alone) [134]. Tremelimumab is another anti-CTLA-4 antibody. In a phase III trial, however, this drug did not show benefit over chemotherapy [136], which suggests that the presence of subtle antibody differences may profoundly affect clinical efficacy. Ipilimumab is generally well tolerated, but has the potential for significant immune-related adverse events (irAEs). Side effects include diarrhea and enterocolitis, hepatitis, dermatitis and endocrinopathies with grade 3 and 4 irAEs occurring in approximately 10%–15% of patients [132,134,137].

Monoclonal antibodies targeted against the immune checkpoint receptor PD-1 have also been developed and approved for use in patients who have progressed on ipilimumab. Lambrolizumab is an anti-PD1 antibody, which in a phase I trial has shown an ORR of up to 52% [138]. Treatment with pembrolizumab, another anti-PD1 antibody showed an ORR of 26% [139]. Lastly, nivolumab was compared to dacarbazine in a recently published phase III randomized trial in previously untreated patients with metastatic melanoma. The one-year OS was 72.9% in the nivolumab group compared to 42.1% in the dacarbazine group, while the median PFS was 5.1 months and 2.2 months, respectively [140]. Due to these promising results, anti-PD-1 therapy has become the *de facto* first-line treatment option for metastatic melanoma.

While these targeted agents have shown benefit for advanced systemic disease, application to extremity IT melanoma in the absence of distant metastases is less well characterized. One small retrospective study evaluated outcomes in patients treated with ipilimumab or IL-2 for disease progression following regional therapy. Fifteen patients treated with IL-2 alone progressed, three out of 12 patients treated with ipilimumab alone had a CR, and three out of six patients treated with IL-2 followed by ipilimumab had a CR [141]. The study concluded that the use of ipilimumab was associated with higher rates of complete response compared to use of IL-2 alone (33 *vs.* 0%; *p* = 0.021) [141].

## 9. Conclusions and Future Directions

The treatment of IT melanoma of the extremity continues to expand and evolve. With the increasing number of therapies available in the armamentarium of IT melanoma, the multimodality approach and sequencing of these novel treatments pose highly interesting areas of investigation. Multiple clinical trials are on-going in an attempt to discover optimal treatment strategies. Importantly, the question of applicability of ipilimumab as adjuvant therapy in patients treated with surgery is being compared to the current standard of IFN in a randomized phase III clinical trial [142]. The results of this trial are eagerly anticipated. Furthermore, ipilimumab is being combined with other therapies, such as intralesional BCG and stereotactic radioablation [142]. Interestingly, ipilimumab is being evaluated as an adjuvant or neoadjuvant approach prior to ILI [18,142]. These are just a few examples of the innovative investigations for IT melanoma which may uncover benefits to combined treatment approaches using both well-established techniques for IT disease and effective systemic immunotherapies.

Regional therapies offer durable responses and potential cure to patients with IT disease. Whereas targeted systemic agents have shown efficacy in distant metastatic disease, their specific application to limited regional extremity disease is less well defined. Direct comparison is difficult given the relative paucity of data specific to systemic agents in the treatment of extremity IT melanoma. On-going trials may help to clarify these issues. Presently, regional therapies have the best response rates and should be considered in patients with unresectable IT disease. Given the nature of ILP and ILI in treating a specific extremity and limiting systemic side effects, use of these regional therapies prior to systemic agents offer unique advantages. When utilizing regional therapies in patients with extremity IT melanoma as the only site of recurrence, the systemic agents can be reserved for use after regional therapy failure or progression of disease to distant metastasis. Also, while the newer immunologic agents may generate a cure and systemic anti-tumor response, their use needs to be balanced by the lower response rates and potential for profound systemic autoimmunity (e.g., panhypopituitarism from hypophysitis). In conclusion, until proven otherwise, ILP and ILI should remain the clinical gold-standard and first-line treatment for isolated IT melanoma, as these regional therapies demonstrate the best responses compared to emerging systemic agents.

## References

[1] SEER Cancer Statistics Factsheets: Melanoma of the Skin. http://seer.cancer.gov/statfacts/html/melan.html.

[2] Edge S.B., Byrd D.R., Compton C.C., Fritz A.G., Greene F.L., Trotti A. (2010). Melanoma of the Skin. American Joint Committee on Cancer Staging Manual.

[3] Read R.L., Haydu L., Saw R.P., Quinn M.J., Shannon K., Spillane A.J., Stretch J.R., Scolyer R.A., Thompson J.F. (2015). In-transit Melanoma Metastases: Incidence, Prognosis, and the Role of Lymphadenectomy. Ann. Surg. Oncol..

[4] Pawlik T.M., Ross M.I., Johnson M.M., Schacherer C.W., McClain D.M., Mansfield P.F., Lee J.E., Cormier J.N., Gershenwald J.E. (2005). Predictors and natural history of in-transit melanoma after sentinel lymphadenectomy. Ann. Surg. Oncol..

[5] Kang J.C., Wanek L.A., Essner R., Faries M.B., Foshag L.J., Morton D.L. (2005). Sentinel lymphadenectomy does not increase the incidence of in-transit metastases in primary melanoma. J. Clin. Oncol..

[6] Beasley G., Tyler D. (2015). In-transit melanoma metastasesncidence, prognosis, and the role of lymphadenectomy. Ann. Surg. Oncol..

[7] Kretschmer L., Beckmann I., Thoms K.M., Mitteldorf C., Bertsch H.P., Neumann C. (2006). Factors predicting the risk of in-transit recurrence after sentinel lymphonodectomy in patients with cutaneous malignant melanoma. Ann. Surg. Oncol..

[8] Stucky C.C., Gray R.J., Dueck A.C., Wasif N., Laman S.D., Sekulic A., Pockaj B.A. (2010). Risk factors associated with local and in-transit recurrence of cutaneous melanoma. Am. J. Surg..

[9] Egger M.E., Tabler B.L., Dunki-Jacobs E.M., Callender G.G., Scoggins C.R., Martin R.C., Quillo A.R., Stromberg A.J., McMasters K.M. (2012). Clinicopathologic and survival differences between upper and lower extremity melanomas. Am. Surg..

[10] Balch C.M., Gershenwald J.E., Soong S.J., Thompson J.F., Atkins M.B., Byrd D.R., Buzaid A.C., Cochran A.J., Coit D.G., Ding S. (2009). Final version of 2009 AJCC melanoma staging and classification. J. Clin. Oncol..

[11] Dong X.D., Tyler D., Johnson J.L., DeMatos P., Seigler H.F. (2000). Analysis of prognosis and disease progression after local recurrence of melanoma. Cancer.

[12] Suojarvi N.J., Jahkola T.A., Virolainen S., Ilmonen S.K., Hernberg M.M. (2012). Outcome following local recurrence or in-transit metastases in cutaneous melanoma. Melanoma Res..

[13] Zager J.S., Puleo C.A., Sondak V.K. (2011). What is the significance of the in transit or interval sentinel node in melanoma?. Ann. Surg. Oncol..

[14] National Comprehensive Cancer Network Melanoma (Version 4.2014). http://www.nccn.org/professionals/physician_gls/pdf/melanoma.pdf.

[15] Abbott A.M., Zager J.S. (2014). Locoregional therapies in melanoma. Surg. Clin. N. Am..

[16] Gimbel M.I., Delman K.A., Zager J.S. (2008). Therapy for unresectable recurrent and in-transit extremity melanoma. Cancer Control.

[17] Hayes A.J., Clark M.A., Harries M., Thomas J.M. (2004). Management of in-transit metastases from cutaneous malignant melanoma. Br. J. Surg..

[18] (2015). Addition of Ipilimumab (MDX-010) To Isolated Limb Infusion (ILI) with Standard Melphalan and Dactinomycin in the Treatment of Advanced Unresectable Melanoma of The Extremity. http://clinicaltrials.gov/show/NCT01323517.

[19] (2015). MDX-010 Antibody, MDX-1379 Melanoma Vaccine, or MDX-010/MDX-1379 Combination Treatment for Patients With Unresectable or Metastatic Melanoma. http://clinicaltrials.gov/show/NCT00094653.

[20] Grotz T.E., Mansfield A.S., Kottschade L.A., Erickson L.A., Otley C.C., Markovic S.N., Jakub J.W. (2011). In-transit melanoma: An individualized approach. Oncology.

[21] Thomas J.M., Clark M.A. (2004). Selective lymphadenectomy in sentinel node-positive patients may increase the risk of local/in-transit recurrence in malignant melanoma. Eur. J. Surg. Oncol..

[22] Estourgie S.H., Nieweg O.E., Kroon B.B. (2004). High incidence of in-transit metastases after sentinel node biopsy in patients with melanoma. Br. J. Surg..

[23] Morton D.L., Thompson J.F., Cochran A.J., Mozzillo N., Nieweg O.E., Roses D.F., Hoekstra H.J., Karakousis C.P., Puleo C.A., Coventry B.J. (2014). Final trial report of sentinel-node biopsy *versus* nodal observation in melanoma. N. Engl. J. Med..

[24] Van Poll D., Thompson J.F., Colman M.H., McKinnon J.G., Saw R.P., Stretch J.R., Scolyer R.A., Uren R.F. (2005). A sentinel node biopsy does not increase the incidence of in-transit metastasis in patients with primary cutaneous melanoma. Ann. Surg. Oncol..

[25] Fincher T.R., McCarty T.M., Fisher T.L., Preskitt J.T., Lieberman Z.H., Stephens J.F., O’Brien J.C., Kuhn J.A. (2003). Patterns of recurrence after sentinel lymph node biopsy for cutaneous melanoma. Am. J. Surg..

[26] Beasley G.M., Speicher P., Sharma K., Seigler H., Salama A., Mosca P., Tyler D.S. (2014). Efficacy of repeat sentinel lymph node biopsy in patients who develop recurrent melanoma. J. Am. Coll. Surg..

[27] Yao K.A., Hsueh E.C., Essner R., Foshag L.J., Wanek L.A., Morton D.L. (2003). Is sentinel lymph node mapping indicated for isolated local and in-transit recurrent melanoma?. Ann. Surg..

[28] (2015). Multicenter Selective Lymphadenectomy Trial II (MSLT-II). http://clinicaltrials.gov/show/NCT00297895.

[29] Sloot S., Rashid O.M., Zager J.S. (2014). Intralesional therapy for metastatic melanoma. Expert Opin. Pharmacother..

[30] Testori A., Faries M.B., Thompson J.F., Pennacchioli E., Deroose J.P., van Geel A.N., Verhoef C., Verrecchia F., Soteldo J. (2011). Local and intralesional therapy of in-transit melanoma metastases. J. Surg. Oncol..

[31] Schon M.P., Schon M. (2007). Imiquimod: Mode of action. Br. J. Dermatol..

[32] Naylor M.F., Crowson N., Kuwahara R., Teague K., Garcia C., Mackinnis C., Haque R., Odom C., Jankey C., Cornelison R.L. (2003). Treatment of lentigo maligna with topical imiquimod. Br. J. Dermatol..

[33] Powell A.M., Russell-Jones R., Barlow R.J. (2004). Topical imiquimod immunotherapy in the management of lentigo maligna. Clin. Exp. Dermatol..

[34] Bong A.B., Bonnekoh B., Franke I., Schon M., Ulrich J., Gollnick H. (2002). Imiquimod, a topical immune response modifier, in the treatment of cutaneous metastases of malignant melanoma. Dermatology.

[35] Kidner T.B., Morton D.L., Lee D.J., Hoban M., Foshag L.J., Turner R.R., Faries M.B. (2012). Combined intralesional Bacille Calmette-Guerin (BCG) and topical imiquimod for in-transit melanoma. J. Immunother..

[36] Tan J.K., Ho V.C. (1993). Pooled analysis of the efficacy of bacille Calmette-Guerin (BCG) immunotherapy in malignant melanoma. J. Dermatol. Surg. Oncol..

[37] Von Wussow P., Block B., Hartmann F., Deicher H. (1988). Intralesional interferon-alpha therapy in advanced malignant melanoma. Cancer.

[38] Hassan S., Petrella T.M., Zhang T., Kamel-Reid S., Nordio F., Baccarelli A., Sade S., Naert K., Habeeb A.A., Ghazarian D. (2015). Pathologic Complete Response to Intralesional Interleukin-2 Therapy Associated with Improved Survival in Melanoma Patients with in-Transit Disease. Ann. Surg. Oncol..

[39] Byers B.A., Temple-Oberle C.F., Hurdle V., McKinnon J.G. (2014). Treatment of in-transit melanoma with intra-lesional interleukin-2, a systematic review. J. Surg. Oncol..

[40] Temple-Oberle C.F., Byers B.A., Hurdle V., Fyfe A., McKinnon J.G. (2014). Intra-lesional interleukin-2 therapy for in transit melanoma. J. Surg. Oncol..

[41] Ross M.I. (2014). Intralesional therapy with PV-10 (Rose Bengal) for in-transit melanoma. J. Surg. Oncol..

[42] Toomey P., Kodumudi K., Weber A., Kuhn L., Moore E., Sarnaik A.A., Pilon-Thomas S. (2013). Intralesional injection of rose bengal induces a systemic tumor-specific immune response in murine models of melanoma and breast cancer. PLoS ONE.

[43] Ito A., Watanabe H., Naito M., Aoyama H., Nakagawa Y., Fujimoto N. (1986). Induction of thyroid tumors in (C_57_BL/6N × C_3_H/N)F_1_ mice by oral administration of 9-3′,4′,5′,6′-tetrachloro-o-carboxy phenyl-6-hydroxy-2,4,5,7-tetraiodo-3-isoxanthone sodium (Food Red 105, rose bengal B). J. Natl. Cancer Inst..

[44] Thompson J.F., Hersey P., Wachter E. (2008). Chemoablation of metastatic melanoma using intralesional Rose Bengal. Melanoma Res..

[45] Foote M.C., Burmeister B.H., Thomas J., Mark Smithers B. (2010). A novel treatment for metastatic melanoma with intralesional rose bengal and radiotherapy: A case series. Melanoma Res..

[46] Thompson J.F., Agarwala S.S., Smithers B.M., Ross M.I., Scoggins C.R., Coventry B.J., Neuhaus S.J., Minor D.R., Singer J.M., Wachter E.A. (2015). Phase 2 Study of Intralesional PV-10 in Refractory Metastatic Melanoma. Ann. Surg. Oncol..

[47] Bartlett D.L., Liu Z., Sathaiah M., Ravindranathan R., Guo Z., He Y., Guo Z.S. (2013). Oncolytic viruses as therapeutic cancer vaccines. Mol. Cancer.

[48] Schipper H., Alla V., Meier C., Nettelbeck D.M., Herchenroder O., Putzer B.M. (2014). Eradication of metastatic melanoma through cooperative expression of RNA-based HDAC1 inhibitor and p73 by oncolytic adenovirus. Oncotarget.

[49] Jiang G., Yang C.S., Xu D., Sun C., Zheng J.N., Lei T.C., Liu Y.Q. (2014). Potent anti-tumour activity of a novel conditionally replicating adenovirus for melanoma via inhibition of migration and invasion. Br. J. Cancer.

[50] Kyula J.N., Khan A.A., Mansfield D., Karapanagiotou E.M., McLaughlin M., Roulstone V., Zaidi S., Pencavel T., Touchefeu Y., Seth R. (2014). Synergistic cytotoxicity of radiation and oncolytic Lister strain vaccinia in (V600D/E)BRAF mutant melanoma depends on JNK and TNF-alpha signaling. Oncogene.

[51] Adamina M., Rosenthal R., Weber W.P., Frey D.M., Viehl C.T., Bolli M., Huegli R.W., Jacob A.L., Heberer M., Oertli D. (2010). Intranodal immunization with a vaccinia virus encoding multiple antigenic epitopes and costimulatory molecules in metastatic melanoma. Mol. Ther..

[52] Zajac P., Oertli D., Marti W., Adamina M., Bolli M., Guller U., Noppen C., Padovan E., Schultz-Thater E., Heberer M. (2003). Phase I/II clinical trial of a nonreplicative vaccinia virus expressing multiple HLA-A0201-restricted tumor-associated epitopes and costimulatory molecules in metastatic melanoma patients. Hum. Gene Ther..

[53] Hwang T.H., Moon A., Burke J., Ribas A., Stephenson J., Breitbach C.J., Daneshmand M., De Silva N., Parato K., Diallo J.S. (2011). A mechanistic proof-of-concept clinical trial with JX-594, a targeted multi-mechanistic oncolytic poxvirus, in patients with metastatic melanoma. Mol. Ther..

[54] (2015). A Study of Recombinant Vaccinia Virus to Treat Malignant Melanoma. http://clinicaltrials.gov/show/NCT00429312.

[55] (2015). Monoclonal Antibody and Vaccine Therapy in Treating Patients With Stage III or Stage IV Melanoma That Has Been Removed During Surgery. http://clinicaltrials.gov/show/NCT00025181.

[56] Kaufman H.L., Collichio F.A., Amatruda T., Senzer N.N., Chesney J., Delman K.A., Spitler L.E., Puzanov I., Ye Y., Li A. (2014). Primary overall survival (OS) from OPTiM, a randomized phase III trial of talimogene laherparepvec (T-VEC) *versus* subcutaneous (SC) granulocyte-macrophage colony-stimulating factor (GM-CSF) for the treatment (tx) of unresected stage IIIB/C and IV melanoma. J. Clin. Oncol..

[57] Andtbacka R.H., Collichio F.A., Amatruda T., Senzer N.N., Chesney J., Delman K.A., Spitler L.E., Puzanov I., Doleman S., Ye Y. (2013). OPTiM: A randomized phase III trial of talimogene laherparepvec (T-VEC) *versus* subcutaneous (SC) granulocyte-macrophage colony-stimulating factor (GM-CSF) for the treatment (tx) of unresected stage IIIB/C and IV melanoma. J. Clin. Oncol..

[58] Andtbacka R.H.I., Ross M.I., Delman K., Dirk Noyes R., Zager J.S., Hsueh E., Ollola D.W., Amatruda T., Chen L., VanderWalde A. (2014). Responses of injected and uninjected lesions to intralesional Talimogene Laherparepvec (T-VEC) in the OPTiM study and the contribution of surgery to response. Ann. Surg. Oncol..

[59] (2015). Ipilimumab With or Without Talimogene Laherparepvec in Unresected Melanoma. http://clinicaltrials.gov/show/NCT01740297.

[60] Guadagnolo B.A., Prieto V., Weber R., Ross M.I., Zagars G.K. (2014). The role of adjuvant radiotherapy in the local management of desmoplastic melanoma. Cancer.

[61] Ballo M.T., Strom E.A., Zagars G.K., Bedikian A.Y., Prieto V.G., Mansfield P.F., Lee J.E., Gershenwald J.E., Ross M.I. (2002). Adjuvant irradiation for axillary metastases from malignant melanoma. Int. J. Radiat. Oncol. Biol. Phys..

[62] Testori A., Rutkowski P., Marsden J., Bastholt L., Chiarion-Sileni V., Hauschild A., Eggermont A.M. (2009). Surgery and radiotherapy in the treatment of cutaneous melanoma. Ann. Oncol..

[63] Mendenhall W.M., Shaw C., Amdur R.J., Kirwan J., Morris C.G., Werning J.W. (2013). Surgery and adjuvant radiotherapy for cutaneous melanoma considered high-risk for local-regional recurrence. Am. J. Otolaryngol..

[64] Seegenschmiedt M.H., Keilholz L., Altendorf-Hofmann A., Pieritz A., Urban A., Schell H., Hohenberger W., Sauer R. (1999). Long term results following radiation therapy of locally recurrent and metastatic malignant melanoma. Hautarzt.

[65] Seegenschmiedt M.H., Keilholz L., Altendorf-Hofmann A., Urban A., Schell H., Hohenberger W., Sauer R. (1999). Palliative radiotherapy for recurrent and metastatic malignant melanoma: Prognostic factors for tumor response and long-term outcome: A 20-year experience. Int. J. Radiat. Oncol. Biol. Phys..

[66] Olivier K.R., Schild S.E., Morris C.G., Brown P.D., Markovic S.N. (2007). A higher radiotherapy dose is associated with more durable palliation and longer survival in patients with metastatic melanoma. Cancer.

[67] Farges O., Fuks D., Boleslawski E., Le Treut Y.P., Castaing D., Laurent A., Ducerf C., Rivoire M., Bachellier P., Chiche L. (2011). Influence of surgical margins on outcome in patients with intrahepatic cholangiocarcinoma: A multicenter study by the AFC-IHCC-2009 study group. Ann. Surg..

[68] Morris K.T., Marquez C.M., Holland J.M., Vetto J.T. (2000). Prevention of local recurrence after surgical debulking of nodal and subcutaneous melanoma deposits by hypofractionated radiation. Ann. Surg. Oncol..

[69] Creech O., Krementz E.T. (1964). Regional perfusion in melanoma of limbs. JAMA.

[70] Alexander H.R., Fraker D.L., Bartlett D.L. (1996). Isolated limb perfusion for malignant melanoma. Semin. Surg. Oncol..

[71] Rossi C.R., Foletto M., Pilati P., Mocellin S., Lise M. (2002). Isolated limb perfusion in locally advanced cutaneous melanoma. Semin. Oncol..

[72] Beasley G.M., Petersen R.P., Yoo J., McMahon N., Aloia T., Petros W., Sanders G., Cheng T.Y., Pruitt S.K., Seigler H. (2008). Isolated limb infusion for in-transit malignant melanoma of the extremity: A well-tolerated but less effective alternative to hyperthermic isolated limb perfusion. Ann. Surg. Oncol..

[73] McDermott P., Lawson D.S., Walczak R., Tyler D., Shearer I.R. (2005). An isolated limb infusion technique: A guide for the perfusionist. J. Extra Corpor. Technol..

[74] Cheng T.Y., Grubbs E., Abdul-Wahab O., Leu S.Y., Hung C.F., Petros W., Aloia T., Fedrau R., Pruitt S., Colvin M. (2003). Marked variability of melphalan plasma drug levels during regional hyperthermic isolated limb perfusion. Am. J. Surg..

[75] Roberts M.S., Wu Z.Y., Siebert G.A., Anissimov Y.G., Thompson J.F., Smithers B.M. (2001). Pharmacokinetics and pharmacodynamics of melphalan in isolated limb infusion for recurrent localized limb malignancy. Melanoma Res..

[76] Kroon H.M., Huismans A., Waugh R.C., Kam P.C., Thompson J.F. (2014). Isolated limb infusion: Technical aspects. J. Surg. Oncol..

[77] Kroon H.M. (2011). Treatment of locally advanced melanoma by isolated limb infusion with cytotoxic drugs. J. Skin Cancer.

[78] Lidsky M.E., Speicher P.J., Jiang B., Tsutsui M., Tyler D.S. (2014). Isolated limb infusion as a model to test new agents to treat metastatic melanoma. J. Surg. Oncol..

[79] Defty C.L., Marsden J.R. (2012). Melphalan in regional chemotherapy for locally recurrent metastatic melanoma. Curr. Top. Med. Chem..

[80] Deroose J.P., Eggermont A.M., van Geel A.N., de Wilt J.H., Burger J.W., Verhoef C. (2012). 20 years experience of TNF-based isolated limb perfusion for in-transit melanoma metastases: TNF dose matters. Ann. Surg. Oncol..

[81] Rossi C.R., Pasquali S., Mocellin S., Vecchiato A., Campana L.G., Pilati P., Zanon A., Nitti D. (2010). Long-term results of melphalan-based isolated limb perfusion with or without low-dose TNF for in-transit melanoma metastases. Ann. Surg. Oncol..

[82] Lienard D., Eggermont A.M., Koops H.S., Kroon B., Towse G., Hiemstra S., Schmitz P., Clarke J., Steinmann G., Rosenkaimer F. (1999). Isolated limb perfusion with tumour necrosis factor-alpha and melphalan with or without interferon-gamma for the treatment of in-transit melanoma metastases: A multicentre randomized phase II study. Melanoma Res..

[83] Kam P.C., Thompson J.F. (2010). Isolated limb infusion with melphalan and actinomycin D in melanoma patients: Factors predictive of acute regional toxicity. Expert Opin. Drug Metab. Toxicol..

[84] Beasley G.M., McMahon N., Sanders G., Augustine C.K., Selim M.A., Peterson B., Norris R., Peters W.P., Ross M.I., Tyler D.S. (2009). A phase 1 study of systemic ADH-1 in combination with melphalan via isolated limb infusion in patients with locally advanced in-transit malignant melanoma. Cancer.

[85] Beasley G.M., Riboh J.C., Augustine C.K., Zager J.S., Hochwald S.N., Grobmyer S.R., Peterson B., Royal R., Ross M.I., Tyler D.S. (2011). Prospective multicenter phase II trial of systemic ADH-1 in combination with melphalan via isolated limb infusion in patients with advanced extremity melanoma. J. Clin. Oncol..

[86] Beasley G.M., Speicher P., Augustine C.K., Dolber P.C., Peterson B.L., Sharma K., Mosca P.J., Royal R., Ross M., Zager J.S. (2015). A multicenter phase I dose escalation trial to evaluate safety and tolerability of intra-arterial temozolomide for patients with advanced extremity melanoma using normothermic isolated limb infusion. Ann. Surg. Oncol..

[87] Raymond A.K., Beasley G.M., Broadwater G., Augustine C.K., Padussis J.C., Turley R., Peterson B., Seigler H., Pruitt S.K., Tyler D.S. (2011). Current trends in regional therapy for melanoma: Lessons learned from 225 regional chemotherapy treatments between 1995 and 2010 at a single institution. J. Am. Coll. Surg..

[88] Olofsson R., Mattsson J., Lindner P. (2013). Long-term follow-up of 163 consecutive patients treated with isolated limb perfusion for in-transit metastases of malignant melanoma. Int. J. Hyperth..

[89] Olofsson Bagge R., Mattsson J., Hafstrom L. (2014). Regional hyperthermic perfusion with melphalan after surgery for recurrent malignant melanoma of the extremities—Long-term follow-up of a randomised trial. Int. J. Hyperth..

[90] Kroon H.M., Moncrieff M., Kam P.C., Thompson J.F. (2008). Outcomes following isolated limb infusion for melanoma. A 14-year experience. Ann. Surg. Oncol..

[91] Kroon H.M., Lin D.Y., Kam P.C., Thompson J.F. (2009). Safety and efficacy of isolated limb infusion with cytotoxic drugs in elderly patients with advanced locoregional melanoma. Ann. Surg..

[92] Beasley G.M., Caudle A., Petersen R.P., McMahon N.S., Padussis J., Mosca P.J., Zager J.S., Hochwald S.N., Grobmyer S.R., Delman K.A. (2009). A multi-institutional experience of isolated limb infusion: Defining response and toxicity in the U.S. J. Am. Coll. Surg..

[93] Deroose J.P., Grunhagen D.J., van Geel A.N., de Wilt J.H., Eggermont A.M., Verhoef C. (2011). Long-term outcome of isolated limb perfusion with tumour necrosis factor-α for patients with melanoma in-transit metastases. Br. J. Surg..

[94] Hoekstra H.J., Veerman K., van Ginkel R.J. (2014). Isolated limb perfusion for in-transit melanoma metastases: Melphalan or TNF-melphalan perfusion?. J. Surg. Oncol..

[95] Cornett W.R., McCall L.M., Petersen R.P., Ross M.I., Briele H.A., Noyes R.D., Sussman J.J., Kraybill W.G., Kane J.M., Alexander H.R. (2006). Randomized multicenter trial of hyperthermic isolated limb perfusion with melphalan alone compared with melphalan plus tumor necrosis factor: American College of Surgeons Oncology Group Trial Z0020. J. Clin. Oncol..

[96] Alexander H.R., Fraker D.L., Bartlett D.L., Libutti S.K., Steinberg S.M., Soriano P., Beresnev T. (2010). Analysis of factors influencing outcome in patients with in-transit malignant melanoma undergoing isolated limb perfusion using modern treatment parameters. J. Clin. Oncol..

[97] Barbour A.P., Thomas J., Suffolk J., Beller E., Smithers B.M. (2009). Isolated limb infusion for malignant melanoma: Predictors of response and outcome. Ann. Surg. Oncol..

[98] Di Filippo F., Giacomini P., Rossi C.R., Santinami M., Anza M., Garinei R., Perri P., Botti C., di Angelo P., Sofra C. (2009). Prognostic factors influencing tumor response, locoregional control and survival, in melanoma patients with multiple limb in-transit metastases treated with TNFalpha-based isolated limb perfusion. In Vivo.

[99] Klaase J.M., Kroon B.B., van Geel A.N., Eggermont A.M., Franklin H.R., Hart A.A. (1994). Prognostic factors for tumor response and limb recurrence-free interval in patients with advanced melanoma of the limbs treated with regional isolated perfusion with melphalan. Surgery.

[100] Muilenburg D.J., Beasley G.M., Thompson Z.J., Lee J.H., Tyler D.S., Zager J.S. (2015). Burden of disease predicts response to isolated limb infusion with melphalan and actinomycin D in melanoma. Ann. Surg. Oncol..

[101] Sharma K., Beasley G., Turley R., Raymond A.K., Broadwater G., Peterson B., Mosca P., Tyler D. (2012). Patterns of recurrence following complete response to regional chemotherapy for in-transit melanoma. Ann. Surg. Oncol..

[102] Lidsky M.E., Turley R.S., Beasley G.M., Sharma K., Tyler D.S. (2013). Predicting disease progression after regional therapy for in-transit melanoma. JAMA Surg..

[103] Chai C.Y., Deneve J.L., Beasley G.M., Marzban S.S., Chen Y.A., Rawal B., Grobmyer S.R., Hochwald S.N., Tyler D.S., Zager J.S. (2012). A multi-institutional experience of repeat regional chemotherapy for recurrent melanoma of extremities. Ann. Surg. Oncol..

[104] Wong J., Chen Y.A., Fisher K.J., Beasley G.M., Tyler D.S., Zager J.S. (2014). Resection of residual disease after isolated limb infusion (ILI) is equivalent to a complete response after ILI-alone in advanced extremity melanoma. Ann. Surg. Oncol..

[105] Wieberdink J., Benckhuysen C., Braat R.P., van Slooten E.A., Olthuis G.A. (1982). Dosimetry in isolation perfusion of the limbs by assessment of perfused tissue volume and grading of toxic tissue reactions. Eur. J. Cancer Clin. Oncol..

[106] Klaase J.M., Kroon B.B., Benckhuijsen C., van Geel A.N., Albus-Lutter C.E., Wieberdink J. (1989). Results of regional isolation perfusion with cytostatics in patients with soft tissue tumors of the extremities. Cancer.

[107] Kroon H.M., Moncrieff M., Kam P.C., Thompson J.F. (2009). Factors predictive of acute regional toxicity after isolated limb infusion with melphalan and actinomycin D in melanoma patients. Ann. Surg. Oncol..

[108] Jiang B.S., Speicher P.J., Thomas S., Mosca P.J., Abernethy A.P., Tyler D.S. Quality of Life After Isolated Limb Infusion for In-Transit Melanoma of the Extremity. Ann. Surg. Oncol..

[109] Pace M., Gattai R., Matteini M., Mascitelli E.M., Bechi P. (2008). Toxicity and morbility after isolated lower limb perfusion in 242 chemo-hyperthermal treatments for cutaneous melanoma: The experience of the Tuscan Reference Centre. J. Exp. Clin. Cancer Res..

[110] Kroon H.M., Lin D.Y., Kam P.C., Thompson J.F. (2009). Major amputation for irresectable extremity melanoma after failure of isolated limb infusion. Ann. Surg. Oncol..

[111] Creagan E.T., Dalton R.J., Ahmann D.L., Jung S.H., Morton R.F., Langdon R.M., Kugler J., Rodrigue L.J. (1995). Randomized, surgical adjuvant clinical trial of recombinant interferon alfa-2a in selected patients with malignant melanoma. J. Clin. Oncol..

[112] Kirkwood J.M., Ibrahim J.G., Sosman J.A., Sondak V.K., Agarwala S.S., Ernstoff M.S., Rao U. (2001). High-dose interferon alfa-2b significantly prolongs relapse-free and overall survival compared with the GM2-KLH/QS-21 vaccine in patients with resected stage IIB-III melanoma: Results of intergroup trial E1694/S9512/C509801. J. Clin. Oncol..

[113] Chiarion-Sileni V., Del Bianco P., Romanini A., Guida M., Paccagnella A., Dalla Palma M., Naglieri E., Ridolfi R., Silvestri B., Michiara M. (2006). Tolerability of intensified intravenous interferon alfa-2b *versus* the ECOG 1684 schedule as adjuvant therapy for stage III melanoma: A randomized phase III Italian Melanoma Inter-group trial (IMI-Mel.A.) [ISRCTN75125874]. BMC Cancer.

[114] Atkins M.B., Hsu J., Lee S., Cohen G.I., Flaherty L.E., Sosman J.A., Sondak V.K., Kirkwood J.M. (2008). Phase III trial comparing concurrent biochemotherapy with cisplatin, vinblastine, dacarbazine, interleukin-2, and interferon alfa-2b with cisplatin, vinblastine, and dacarbazine alone in patients with metastatic malignant melanoma (E3695): A trial coordinated by the Eastern Cooperative Oncology Group. J. Clin. Oncol..

[115] Sharma A., Shah S.R., Illum H., Dowell J. (2012). Vemurafenib: Targeted inhibition of mutated BRAF for treatment of advanced melanoma and its potential in other malignancies. Drugs.

[116] Khoja L., Hogg D. (2015). Dabrafenib in the treatment of metastatic or unresectable melanoma. Expert Rev. Anticancer Ther..

[117] Ascierto P.A., Kirkwood J.M., Grob J.J., Simeone E., Grimaldi A.M., Maio M., Palmieri G., Testori A., Marincola F.M., Mozzillo N. (2012). The role of BRAF V600 mutation in melanoma. J. Transl. Med..

[118] McDermott D., Lebbe C., Hodi F.S., Maio M., Weber J.S., Wolchok J.D., Thompson J.A., Balch C.M. (2014). Durable benefit and the potential for long-term survival with immunotherapy in advanced melanoma. Cancer Treat. Rev..

[119] Hauschild A., Grob J.J., Demidov L.V., Jouary T., Gutzmer R., Millward M., Rutkowski P., Blank C.U., Miller W.H., Kaempgen E. (2012). Dabrafenib in BRAF-mutated metastatic melanoma: A multicentre, open-label, phase 3 randomised controlled trial. Lancet.

[120] Solit D.B., Rosen N. (2011). Resistance to BRAF inhibition in melanomas. N. Engl. J. Med..

[121] Gilmartin A.G., Bleam M.R., Groy A., Moss K.G., Minthorn E.A., Kulkarni S.G., Rominger C.M., Erskine S., Fisher K.E., Yang J. (2011). GSK1120212 (JTP-74057) is an inhibitor of MEK activity and activation with favorable pharmacokinetic properties for sustained *in vivo* pathway inhibition. Clin. Cancer Res..

[122] Robert C., Karaszewska B., Schachter J., Rutkowski P., Mackiewicz A., Stroiakovski D., Lichinitser M., Dummer R., Grange F., Mortier L. (2015). Improved overall survival in melanoma with combined dabrafenib and trametinib. N. Engl. J. Med..

[123] Johnson D.B., Flaherty K.T., Weber J.S., Infante J.R., Kim K.B., Kefford R.F., Hamid O., Schuchter L., Cebon J., Sharfman W.H. (2014). Combined BRAF (Dabrafenib) and MEK inhibition (Trametinib) in patients with BRAFV600-mutant melanoma experiencing progression with single-agent BRAF inhibitor. J. Clin. Oncol..

[124] Long G.V., Stroyakovskiy D., Gogas H., Levchenko E., de Braud F., Larkin J., Garbe C., Jouary T., Hauschild A., Grob J.J. (2014). Combined BRAF and MEK inhibition *versus* BRAF inhibition alone in melanoma. N. Engl. J. Med..

[125] Peters S., Bouchaab H., Zimmerman S., Bucher M., Gaide O., Letovanec I., Homicsko K., Michielin O. (2014). Dramatic response of vemurafenib-induced cutaneous lesions upon switch to dual BRAF/MEK inhibition in a metastatic melanoma patient. Melanoma Res..

[126] Long G.V., Stroyakovskiy D., Gogas H., Levchenko E., de Braud F., Larkin J., Garbe C., Jouary T., Hauschild A., Grob J.J. Dabrafenib and trametinib *versus* dabrafenib and placebo for Val600 BRAF-mutant melanoma: A multicentre, double-blind, phase 3 randomised controlled trial. Lancet.

[127] Seremet T., Lienard D., Suppa M., Trepant A.L., Rorive S., Woff E., Cuylits N., Jansen Y., Schreuer M., Del Marmol V. (2015). Successful (neo)adjuvant BRAF-targeted therapy in a patient with locally advanced BRAF V600E mutant melanoma. Melanoma Res..

[128] Dossett L.A., Kudchadkar R.R., Zager J.S. (2015). BRAF and MEK inhibition in melanoma. Expert Opin. Drug Saf..

[129] Menzies A.M., Long G.V. (2014). Dabrafenib and trametinib, alone and in combination for BRAF-mutant metastatic melanoma. Clin. Cancer Res..

[130] Larkin J., del Vecchio M., Ascierto P.A., Krajsova I., Schachter J., Neyns B., Espinosa E., Garbe C., Sileni V.C., Gogas H. (2014). Vemurafenib in patients with BRAF(V600) mutated metastatic melanoma: An open-label, multicentre, safety study. Lancet Oncol..

[131] Gabriel E.M., Lattime E.C. (2007). Anti-CTL-associated antigen 4, are regulatory T cells a target?. Clin. Cancer Res..

[132] Hodi F.S., O’Day S.J., McDermott D.F., Weber R.W., Sosman J.A., Haanen J.B., Gonzalez R., Robert C., Schadendorf D., Hassel J.C. (2010). Improved survival with ipilimumab in patients with metastatic melanoma. N. Engl. J. Med..

[133] Maio M., Grob J.J., Aamdal S., Bondarenko I., Robert C., Thomas L., Garbe C., Chiarion-Sileni V., Testori A., Chen T.T. Five-Year Survival Rates for Treatment-Naive Patients With Advanced Melanoma Who Received Ipilimumab Plus Dacarbazine in a Phase III Trial. J. Clin. Oncol..

[134] Robert C., Thomas L., Bondarenko I., O’Day S., Weber J., Garbe C., Lebbe C., Baurain J.F., Testori A., Grob J.J. (2011). Ipilimumab plus dacarbazine for previously untreated metastatic melanoma. N. Engl. J. Med..

[135] Sarnaik A.A., Yu B., Yu D., Morelli D., Hall M., Bogle D., Yan L., Targan S., Solomon J., Nichol G. (2011). Extended dose ipilimumab with a peptide vaccine: Immune correlates associated with clinical benefit in patients with resected high-risk stage IIIc/IV melanoma. Clin. Cancer Res..

[136] Ribas A., Kefford R., Marshall M.A., Punt C.J., Haanen J.B., Marmol M., Garbe C., Gogas H., Schachter J., Linette G. (2013). Phase III randomized clinical trial comparing tremelimumab with standard-of-care chemotherapy in patients with advanced melanoma. J. Clin. Oncol..

[137] Wiater K., Switaj T., Mackiewicz J., Kalinka-Warzocha E., Wojtukiewicz M., Szambora P., Falkowski S., Rogowski W., Mackiewicz A., Rutkowski P. (2013). Efficacy and safety of ipilimumab therapy in patients with metastatic melanoma: A retrospective multicenter analysis. Contemp. Oncol..

[138] Hamid O., Robert C., Daud A., Hodi F.S., Hwu W.J., Kefford R., Wolchok J.D., Hersey P., Joseph R.W., Weber J.S. (2013). Safety and tumor responses with lambrolizumab (anti-PD-1) in melanoma. N. Engl. J. Med..

[139] Robert C., Ribas A., Wolchok J.D., Hodi F.S., Hamid O., Kefford R., Weber J.S., Joshua A.M., Hwu W.J., Gangadhar T.C. (2014). Anti-programmed-death-receptor-1 treatment with pembrolizumab in ipilimumab-refractory advanced melanoma: A randomised dose-comparison cohort of a phase 1 trial. Lancet.

[140] Robert C., Long G.V., Brady B., Dutriaux C., Maio M., Mortier L., Hassel J.C., Rutkowski P., McNeil C., Kalinka-Warzocha E. (2015). Nivolumab in previously untreated melanoma without BRAF mutation. N. Engl. J. Med..

[141] Jiang B.S., Beasley G.M., Speicher P.J., Mosca P.J., Morse M.A., Hanks B., Salama A., Tyler D.S. (2014). Immunotherapy following regional chemotherapy treatment of advanced extremity melanoma. Ann. Surg. Oncol..

[142] National Cancer Institute (2015). Ipilimumab or High-Dose Interferon Alfa-2b in Treating Patients With High-Risk Stage III-IV Melanoma That Has Been Removed by Surgery.

